# Exploring the Link Between Stress and Working Memory in Adults

**DOI:** 10.3390/ijerph22121773

**Published:** 2025-11-24

**Authors:** Constança Carvalho, Cláudia Reis, Margarida Serrano

**Affiliations:** 1 Polytechnic University of Coimbra, Rua da Misericórdia, Lagar dos Cortiços, S. Martinho do Bispo, 3045-093 Coimbra, Portugalcreis@estesc.ipc.pt (C.R.); 2 H&TRC—Health & Technology Research Center, Coimbra Health School, Polytechnic University of Coimbra, Rua 5 de Outubro, 3046-854 Coimbra, Portugal

**Keywords:** working memory, auditory memory, perceived stress, pseudoword span, cognitive performance

## Abstract

Background: Working memory (WM) is essential for reasoning, learning, and everyday cognitive tasks and can be influenced by stress. This study investigated the relationship between perceived stress and auditory working memory in 24 adults (16 women, 8 men; median age = 22). Methods: Participants completed the Perceived Stress Scale (PSS-10) and a pseudoword span task assessing auditory memory under phonologically demanding conditions. Results: Participants with higher stress levels exhibited greater variability and a decline in performance across pseudowords sets, particularly in the final set. Correlational analyses revealed that higher PSS-10 scores were significantly associated with lower accuracy on the most demanding memory set (r = −0.467, *p* = 0.021) and with younger age (r = −0.489, *p* = 0.015). These findings suggest that elevated stress may impair auditory working memory, with younger adults reporting higher perceived stress. Conclusions: This study highlights the importance of considering stress levels in cognitive assessments and supports the hypothesis that stress negatively affects working memory efficiency, particularly in tasks requiring phonological processing.

## 1. Introduction

Working memory (WM) is described as the cognitive ability to temporarily retain and manipulate information, enabling the performance of complex tasks despite distraction or interference [1,2,3,4]. Many authors believe it underpins reasoning, comprehension, problem-solving, and academic skills such as mathematics and verbal tasks, and serves as a bridge to long-term memory [5,6,7]. Working Memory Span has been used as a tool to analyze the role of working memory in cognitive skills such as reasoning and learning, however the existence of a link between working memory and intelligence is not perfectly clear, and some authors question to what extent processing and storage are important for intelligence [8,9]. WM has inherent capacity limitations, which can be enhanced through strategies such as rehearsal, chunking, or phonologically demanding activities like letter naming, sentence recall, and story listening [7].

According to the classical model of Baddeley and Hitch (1974), WM comprises the central executive component, which coordinates and manipulates information while directing attention; the phonological loop, responsible for verbal and auditory storage; Visuo-Spatial Sketching, which maintains and manipulates visual–spatial representations; and the episodic buffer, which integrates information across modalities and links WM to long-term memory [10].

Auditory memory, the ability to recall orally presented information, is closely linked to WM and supports verbal comprehension, active listening, and communication [11]. It can be assessed using pseudowords, which minimize lexical and semantic influences. Forward and backward pseudoword span tasks engage the phonological loop and central executive component, while pseudoword N-back tasks measure dynamic updating [12,13]. These tasks complement standard WM assessments, such as complex span tasks, visuospatial tasks, and numerical manipulations, enabling comprehensive evaluation across verbal and visuospatial domains [2,14,15,16,17].

External factors, particularly stress, can influence WM and auditory memory. Stress, defined as mental or physiological tension in response to challenges, activates the hypothalamic–pituitary–adrenal axis and increases cortisol, affecting brain regions crucial for memory and decision-making [18,19,20]. While moderate stress may enhance adaptive responses, chronic or excessive stress impairs WM, attention, and problem-solving. Individual responses vary, with acute stress eliciting rapid survival-oriented reactions and chronic stress producing prolonged cognitive effects [21,22].

Stress can be measured using the Perceived Stress Scale (PSS), a brief, validated self-report tool suitable for clinical, occupational, and educational contexts. There are three versions of the PSS. The original instrument is a 14-item scale (PSS-14) that was developed in English, with seven positive items and seven negative items rated on a five-point Likert scale. Five years after the introduction of the PSS-14, it was shortened to 10 items (PSS-10) using factor analysis based on data from 2387 U.S. residents. A four-item PSS (PSS-4) was also introduced as a brief version for situations requiring a very short scale or telephone interviews. PSS, when combined with physiological indicators or assessments of coping and resilience, allows for the examination of the relationship between perceived stress and adaptive or maladaptive cognitive responses [23,24].

However, the efficiency of the auditory working memory system (AWM) is highly sensitive to emotional and physiological states, particularly stress. Stress activates the hypothalamic–pituitary–adrenal axis and triggers cortisol release, which can modulate neural circuits within the prefrontal cortex and hippocampus, thereby affecting the attentional and executive functions that underpin AWM performance [25,26]. Evidence from behavioral and neuroimaging studies suggests that acute stress can temporarily disrupt auditory information processing and phonological storage, whereas chronic stress leads to longer-term alterations in synaptic plasticity and neural connectivity within auditory and prefrontal regions [27,28]. Understanding the interplay between stress and AWM is therefore essential for elucidating how emotional regulation and environmental pressures influence language comprehension and cognitive performance, particularly during tasks involving phonological complexity or high cognitive load.

This study aims to understand and identify the impact of stress on working memory, and consequently on auditory memory, by analyzing how they are affected, using tasks with phonological complexity.

## 2. Materials and Methods

### 2.1. Ethics

Ethical approval for the study was granted by the Ethics Committee of the Polytechnic Institute of Coimbra (approval number D94/2024) prior to the start of data collection. All participants provided both written and verbal informed consent.

### 2.2. Participants

The sample consisted of twenty-four participants (16 women, 66.7%; 8 men, 33.3%) aged 18 to 59 years (median: 22.0). All participants were native European Portuguese speakers, with type A or C1 tympanograms, and hearing thresholds below 20 dB at 0.5, 1, 2, and 4 kHz in both ears. They were able to write independently and had no known conditions affecting memory.

### 2.3. Procedure

All participants first completed a comprehensive questionnaire to collect demographic information and assess tinnitus, memory performance, and stress exposure. Subsequently, they completed the Perceived Stress Scale (PSS-10), followed by an auditory working memory task to measure phonological memory using the pseudoword span test.

The Perceived Stress Scale (PSS-10), originally developed by Cohen et al. [23,24] and later validated for the Portuguese population by Trigo et al. [29], is a self-report instrument that assesses the degree to which individuals perceive their lives as stressful. The scale captures subjective evaluations of stress, emphasizing psychological interpretation rather than objective exposure. It comprises ten items assessing the frequency of feelings of unpredictability, lack of control, and overload experienced during the past 30 days.

Responses are provided on a five-point Likert scale (0 = never, 1 = almost never, 2 = sometimes, 3 = fairly often, 4 = very often), allowing a detailed assessment of stress frequency. Four items (Items 4, 5, 7, and 8) are reverse scored to reduce acquiescence bias and balance positive and negative appraisals. The total score is obtained by summing all items, yielding a value between 0 and 40, with higher scores indicating greater perceived stress. Scores from 0 to 13 indicate low stress, 14–26 moderate stress, and 27–40 high stress. The Portuguese version demonstrated high internal consistency (Cronbach’s α = 0.874) and robust construct validity, consistent with previous studies reporting α values between 0.78 and 0.91.

In the present study, the PSS-10 was used to quantify subjective stress levels and classify participants into low, moderate, or high stress groups. These classifications were used as the basis for subsequent comparisons of auditory working memory performance.

The classification of participants into low (0–13), moderate (14–26), and high (27–40) stress groups was based on the cutoff criteria established in the original validation study of the PSS-10 by Cohen et al. [23,24] and further supported by the Portuguese adaptation by Trigo et al. [29]. These thresholds are widely adopted in the literature to ensure consistency and comparability across studies assessing perceived stress. They represent empirically derived score ranges corresponding to distinct levels of stress perception in community samples. Given that the present study involved a non-clinical adult population with expected variability in stress exposure, the use of these standardized cutoffs was considered appropriate to capture meaningful differences in perceived stress intensity and to enable group-based comparisons of auditory working memory performance.

Prior to the experimental task, participants underwent a training period phase with the pseudoword span procedure. During this phase, the researcher explained the task and provided several practice trials to ensure that participants clearly understood how to listen, memorize, and repeat the pseudowords in the correct order. Practice data were excluded from the analysis.

The experimental task was conducted in a soundproof audiometric booth at the Audiology Laboratory of the Coimbra Health School under rigorously controlled acoustic conditions to minimize external noise. All individuals were seated alone inside the soundproof boot, and the test was performed using headphones. Participants were presented with four sets of pseudowords, each acoustically degraded by the addition of bubble noise calibrated to a signal-to-noise ratio (SNR) of +10 dB, simulating everyday listening environments. The task was designed to progressively increase cognitive load across sets:

- Set 1: three groups of two pseudowords each (6 pseudowords total). Example: Deima, Nuto;
- Set 2: three groups of three pseudowords (9 total). Example: Têdo, Fisto, Rono;
- Set 3: three groups of four pseudowords (12 total). Example: Bapo, Finha, Zôta, Mage;
- Set 4: three groups of five pseudowords (15 total). Example: Tula, Caufa, Muta, Vinço, Sêto.

All stimuli were presented binaurally through a GSI Audiostar Pro (Grason-Stadler (GSI), MN, USA) audiometer with Radioear DD 65 v2 headphones at a constant intensity level of 50 dB HL. The audiometer was calibrated following standard audiometric procedures.

Participants were instructed to listen carefully to each group of pseudowords, memorize them, and repeat them aloud in the same order. Accuracy was prioritized over response speed. Correctly recalled pseudowords were recorded and scored for each set, providing a quantitative measure of auditory working memory performance under varying phonological and noise conditions. The order of presentation inside each set was randomized.

The entire experimental procedure lasted approximately 20 min.

### 2.4. Statistical Analysis

Descriptive statistics summarized the data. Continuous variables are reported as mean, median, and standard deviation (SD), and categorical variables as counts (n) and percentages (%). Pearson correlation assessed associations between variables, and group differences were evaluated using the T-test. Statistical significance was set at *p* < 0.05. Values of *p* between 0.05 and 0.1 were interpreted as marginally significant.

## 3. Results

Of the 24 participants, 3 (12.5%) reported experiencing tinnitus, 7 (29.2%) indicated having poor memory, 7 (29.2%) considered themselves highly stressed, and 11 (45.8%) reported exposure to high levels of daily stress. Overall, participants exhibited moderate to high stress levels, with a median PSS-10 score of 25 (IQR = 23–28) (Table 1).

No participants fell into the low stress category; 14 (58.3%) were classified as moderate stress, and 10 (41.7%) as high stress. Stress levels were evenly distributed with respect to female participants and median age (Table 2), with the interquartile range of age spanning 19.75–47.25 years in the moderate stress group and 20.75–24.00 years in the high stress group.

Within the moderate stress group (n = 14), PSS-10 scores ranged from 14 to 25 (median = 23.5, mean = 22.57, SD = 2.98), whereas scores in the high stress group (n = 10) ranged from 27 to 32 (median = 28.5, mean = 28.60, SD = 1.58), indicating lower variability among participants with high stress (Table 3). Notably, in the high stress group, 20% (n = 2) reported tinnitus, 70% (n = 7) considered themselves to have good memory, 90% (n = 9) perceived themselves as highly stressed, and 70% (n = 7) reported exposure to high daily stress. In contrast, corresponding values in the moderate stress group were 7.1% (n = 1), 71.4% (n = 10), 57.1% (n = 8), and 28.6% (n = 4).

Descriptive statistics for auditory memory performance across four sets are presented in Table 4. Performance was highest and most consistent in the first set (M = 5.67, SD = 0.48). Variability increased in subsequent sets (SD = 2.26–2.48), with wider score ranges, reflecting less consistent performance. By the fourth set, the mean declined to 4.17, and the minimum score reached 0, indicating a general decrease in performance over time.

Table 5 shows that the moderate stress group maintained more stable outcomes, whereas the high stress group exhibited greater variability and a notable decline in the final set, suggesting that elevated stress may adversely impact auditory memory. No statistically significant differences were observed between groups, except in the fourth set, where the difference between groups was marginally significant (*p* = 0.083).

Correlational analysis revealed that PSS-10 scores were significantly negatively associated with performance on the fourth set (r = −0.467, *p* = 0.021), indicating that higher perceived stress was related to lower accuracy in phonological working memory tasks. Additionally, PSS-10 scores were negatively correlated with age (r = −0.489, *p* = 0.015) (Table 6).

## 4. Discussion

Stress, both acute and chronic, is frequently associated with cognitive challenges, including potential effects on auditory working memory, particularly in tasks requiring verbal information processing and attention allocation [25,30,31]. This study examined perceived stress and its relationship with auditory working memory in adults using a pseudoword span task. Descriptively, participants with higher stress levels tended to show slightly lower performance in the most phonologically demanding sets, though group differences were not statistically significant. The marginal trend observed in the fourth set (*p* = 0.083) should therefore be interpreted cautiously.

The high-stress group had a lower mean age (22.2 years) compared to the moderate-stress group (31.1 years). While younger participants reported slightly higher perceived stress, descriptive patterns suggest that stress, rather than age alone, may be associated with variability in auditory memory performance. Participants with moderate stress generally maintained more stable performance, whereas those with higher stress exhibited greater variability and a descriptive decline in later sets [21,32,33].

Correlational analyses revealed two notable trends. First, higher PSS-10 scores were moderately negatively associated with performance on the fourth pseudoword set (r = −0.467, *p* = 0.021), indicating that participants reporting higher perceived stress tended to perform worse on the most demanding phonological memory task [34]. Second, PSS-10 scores were negatively correlated with age (r = −0.489, *p* = 0.015), suggesting that younger participants tended to report higher perceived stress. While these correlations provide preliminary insight into potential relationships between stress, age, and auditory working memory, they should be interpreted cautiously given the small sample size and cross-sectional design [35].

Physiologically, stress can induce cortisol release, which may impair prefrontal cortex function and disrupt executive processes necessary for maintaining and manipulating verbal information [25,31]. Overall, the descriptive results suggest that auditory working memory may be sensitive to perceived stress, particularly under higher cognitive load [33,36]. Moderate stress appeared compatible with more stable performance, whereas higher stress was associated with greater variability and lower mean performance.

## 5. Conclusions

Descriptive trends from this study suggest that elevated perceived stress may be associated with slightly reduced auditory working memory performance, particularly in tasks with higher phonological complexity. The observed correlations indicate a potential relationship between higher perceived stress and lower performance in demanding memory tasks, as well as a tendency for younger participants to report higher stress levels. However, the small sample size and lack of significant group differences limit generalizability and preclude causal inference.

Preventive strategies, including regular exercise, adequate sleep, relaxation techniques, time management, and social support, may help mitigate potential effects of stress on cognition [37]. Future research with larger, longitudinal samples is needed to confirm these descriptive trends and to examine protective factors, such as resilience or coping strategies, for mitigating stress-related cognitive challenges [38]. Overall, the findings highlight the potential sensitivity of auditory working memory to perceived stress and underscore the importance of early interventions, particularly in younger adults.

## Figures and Tables

**Table 1:** Overall PSS-10 scores for All Participants.

	PSS-10
n	24
Mean	25.08
Median	25.00
Standard Deviation	3.90
Maximum	32.00
Minimum	14.00

**Table 2:** Demographic Characteristics by Stress Group.

		Female	Male	Age (Median/Mean)	Total
Moderate stress	n	8	6	22.5/22.2	14
	%	57.1%	42.9%		58.3%
High stress	n	8	2	22.0/31.1	10
	%	80.0%	20.0%		41.7%
Total	n	16	8	22.0/27.4	24
	%	66.7%	33.3%		100%

**Table 3:** PSS-10 scores by Stress Group.

		PSS-10
Moderate Stress Score 14–26	n	14
	Mean	22.57
	Median	23.50
	Standard Deviation	2.98
	Maximum	25.00
	Minimum	14.00
High Stress Score 27–40	n	10
	Mean	28.60
	Median	28.50
	Standard Deviation	1.58
	Maximum	32.00
	Minimum	27.00

**Table 4:** Descriptive Statistics for Auditory Memory Performance Across Four Sets.

	First Set	Second Set	Third Set	Fourth Set
n	24	24	24	24
Mean	5.67	4.54	5.33	4.17
Median	6.00	5.00	5.00	4.00
Standard Deviation	0.48	2.26	2.48	2.01
Maximum	6.00	9.00	10.00	8.00
Minimum	5.00	1.00	0.00	0.00

**Table 5:** Auditory Memory Performance Across Moderate and High Stress Groups.

		First Set	Second Set	Third Set	Fourth Set
Moderate Stress Score 14–26	n	14	14	14	14
	Mean	5.71	4.43	5.50	4.79
	Median	6.00	4.50	5.00	4.50
	Standard Deviation	0.47	2.28	2.35	1.81
	Maximum	6.00	9.00	10.00	8.00
	Minimum	5.00	1.00	2.00	2.00
High Stress Score 27–40	n	10	10	10	10
	Mean	5.60	4.70	5.10	3.30
	Median	6.00	5.50	5.00	4.00
	Standard Deviation	0.52	2.36	2.77	2.06
	Maximum	6.00	8.00	10.00	7.00
	Minimum	5.00	1.00	0.00	0.00

**Table 6:** Correlations between PSS-10 and Age, and between PSS-10 and Phonological Working Memory Tasks.

		First Set	Second Set	Third Set	Fourth Set	Age
PSS-10	r	−0.170	0.054	−0.057	−0.467	−0.489
	*p*	0.428	0.803	0.791	0.021	0.015
	n	24	24	24	24	24

## Data Availability

Dataset available on request from the authors.
